# Assembly and transport of filovirus nucleocapsids

**DOI:** 10.1371/journal.ppat.1010616

**Published:** 2022-07-28

**Authors:** Olga Dolnik, Stephan Becker

**Affiliations:** Institute of Virology, Philipps-University Marburg, Marburg, Germany; Boston University, UNITED STATES

## Abstract

Filovirus-infected cells are characterized by typical cytoplasmic inclusion bodies (IBs) located in the perinuclear region. The formation of these IBs is induced mainly by the accumulation of the filoviral nucleoprotein NP, which recruits the other nucleocapsid proteins, the polymerase co-factor VP35, the polymerase L, the transcription factor VP30 and VP24 via direct or indirect protein–protein interactions. Replication of the negative-strand RNA genomes by the viral polymerase L and VP35 occurs in the IBs, resulting in the synthesis of positive-strand genomes, which are encapsidated by NP, thus forming ribonucleoprotein complexes (antigenomic RNPs). These newly formed antigenomic RNPs in turn serve as templates for the synthesis of negative-strand RNA genomes that are also encapsidated by NP (genomic RNPs). Still in the IBs, genomic RNPs mature into tightly packed transport-competent nucleocapsids (NCs) by the recruitment of the viral protein VP24. NCs are tightly coiled left-handed helices whose structure is mainly determined by the multimerization of NP at its N-terminus, and these helices form the inner layer of the NCs. The RNA genome is fixed by 2 lobes of the NP N-terminus and is thus guided by individual NP molecules along the turns of the helix. Direct interaction of the NP C-terminus with the VP35 and VP24 molecules forms the outer layer of the NCs. Once formed, NCs that are located at the border of the IBs recruit actin polymerization machinery to one of their ends to drive their transport to budding sites for their envelopment and final release. Here, we review the current knowledge on the structure, assembly, and transport of filovirus NCs.

## 1. Introduction

**Fig 1:**
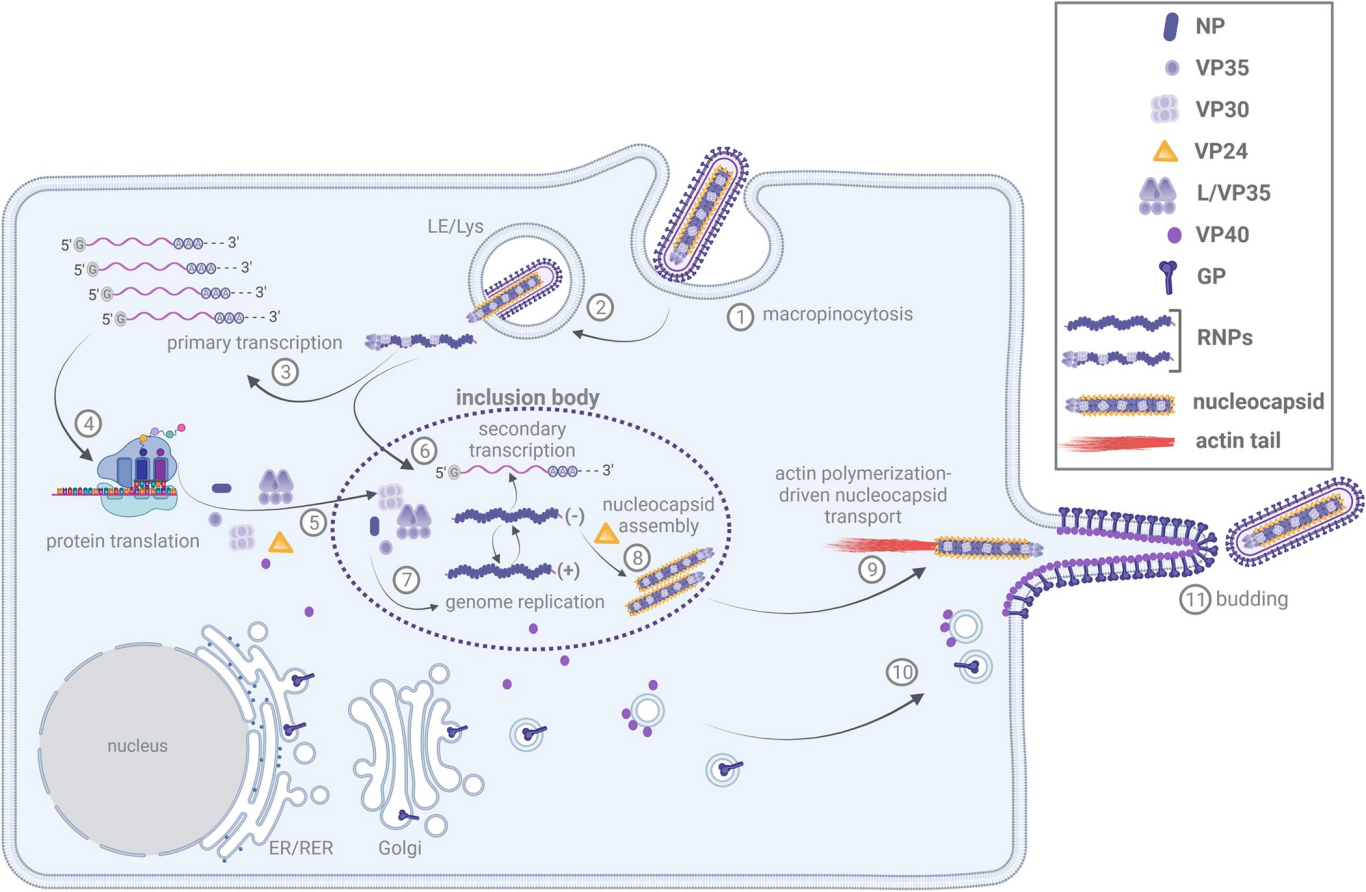
Filovirus replication cycle. Viral entry into target cells is initiated after attachment to the plasma membrane and is accomplished by macropinocytosis (1). During maturation of macropinocytic vesicles into LE/Lys, the viral envelope fuses with the LE/Lys membrane, and the NC is released into the cytosol (2), where it serves as a template for the primary transcription of capped viral mRNAs (3). Translation of viral proteins (4) occurs at free ribosomes, with the exception of GP, which is translated at the rough endoplasmatic reticulum (ER/RER). GP is transported through the classical secretory pathway via the Golgi apparatus to the plasma membrane. The matrix protein VP40 associates with membrane vesicles and is transported to the plasma membrane to form the viral envelope together with GP and cellular lipids (10). Expression of NC proteins (NP, VP35, VP30, VP24, and L) leads to the formation of IBs (5) where secondary transcription (6), genome replication (7), and NC assembly are organized (8). NCs are transported directionally from IBs to budding sites through actin polymerization-driven transport mediated by actin tail formation at one end (9). Envelopment of NCs by membranes containing VP40 and GP occurs at the plasma membrane where budding of filoviruses take place (11). Figure was created with BioRender.com. IB, inclusion body; LE/Lys, late endosomes/lysosomes; RER, rough endoplasmatic reticulum; RNP, ribonucleoprotein complex.

*Ebolavirus* (EBOV) and *Marburgvirus* (MARV) represent 2 genera of the family *Filoviridae*, which includes important zoonotic pathogens. EBOV and MARV cause fatal outbreaks in humans with case fatality rates of up to 90% [1]. Because of their nonsegmented single-stranded RNA genome that has negative polarity, filoviruses belong to the order *Mononegavirales*. Filovirus particles have a characteristic filamentous shape that is approximately 1 μm in length and a diameter of approximately 90 nm. The viral genome is encapsidated by the nucleoprotein NP and is associated with 4 additional viral proteins (VP): VP35 (the analog of the P protein in other mononegaviruses), the polymerase L, the transcription factor VP30 and VP24, forming the nucleocapsid (NC). The matrix protein VP40 surrounds the NC, forming a regular layer beneath the viral envelope. The glycoprotein GP is incorporated into the viral envelope [2]. The entry of viral particles into cells is initiated by the recognition of target cells by GP, and entry is mediated by macropinocytosis (Fig 1) [3,4]. During endocytosis, macropinocytic vesicles mature into late endosomes/lysosomes, where GP is cleaved by cellular proteases. This process exposes the receptor-binding domain of GP to the endosomal receptor NPC1, and receptor binding initiates fusion of the viral and lysosomal membranes, which results in the release of NCs into the cytosol [5]. Here, the NC-associated polymerase complex, which consists of L, the polymerase cofactor VP35, and the transcription initiation factor VP30, initiates the primary transcription of viral mRNAs. Except for GP, which is translated at ER-bound ribosomes and transported through the classical secretory pathway to the plasma membrane, all the other viral proteins are translated at free ribosomes in the cytosol. Increasing amounts of viral proteins in the cytosol lead to the formation of inclusion bodies (IBs), which are a hallmark of filovirus infection [6,7]. IBs represent sites of secondary transcription, genome replication, and de novo NC formation of transport-competent NCs that are transported to budding sites at the plasma membrane for envelopment (Fig 1) [8–12]. In the literature, the term NC is often used equivalently to the term ribonucleoprotein complex (RNP). In this review, we consider RNPs as functional complexes composed of viral RNA, NP, VP35, L, and VP30 that are active in transcription or replication [13]. Therefore, RNPs can have different protein compositions consisting of the viral RNA, NP, VP35, and L with or without the transcription initiation factor VP30. In comparison, NCs are defined as discrete condensed structures that are also detected inside the viral particles composed of the genomic RNA, NP, VP35, L, VP30, and VP24 (Fig 1). The complete replication cycle of filoviruses was reviewed by Kolesnikova and colleagues [14]. Here, we review the current knowledge on the structure, assembly, and transport of filovirus NCs from their assembly site in IBs to the budding sites at the plasma membrane, where they are packaged into filamentous infectious virions.

## 2. Structural components of filovirus nucleocapsids

**Fig 2:**
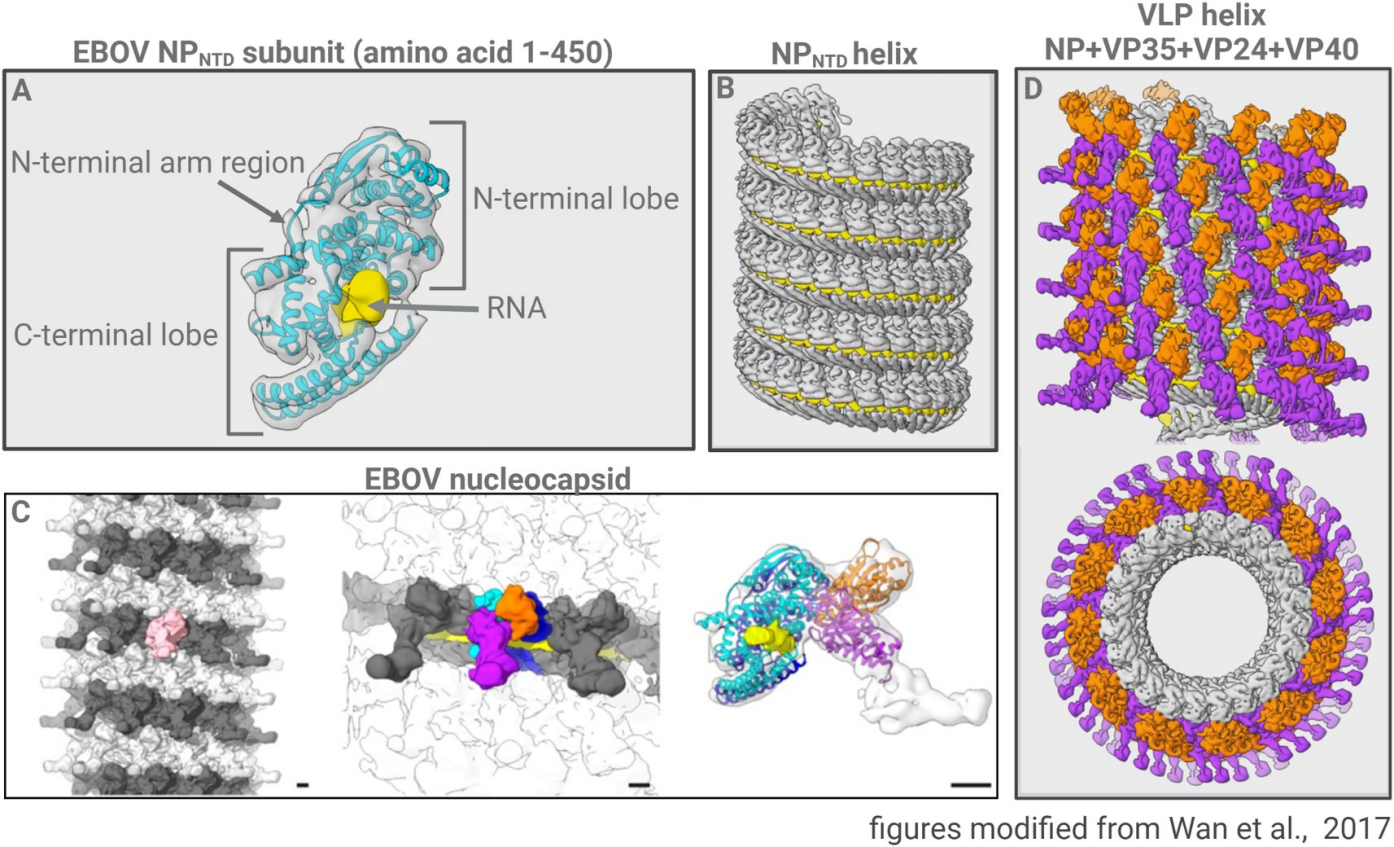
Structure of Ebola virus nucleocapsids [15]. (**A**) NP_NTD_ monomer (amino acid 1–450) derived from subtomogram averaging of NP_NTD_ helices (see B) with the aligned crystal structure in cyan and the putative RNA binding site in yellow. An arrow indicates the N-terminal arm region with the N-terminal helix. (**B**) The left-handed NP_NTD_ helix bound to an RNA chain (yellow). (**C**) Model of the EBOV NC derived from Cryo-ET and subtomogram averaging. Left: NC structure of EBOV. Subunits of 1 rung are shown in dark or light gray. One subunit is highlighted in pink. Middle: The NP_NTD_ subunits are highlighted in cyan and blue, the structures highlighted in orange, and purple are NP_CTD_ protrusions, 2 VP24 molecules and VP35. The bound RNA is highlighted in yellow. Right: side view of the asymmetric protomer showing 2 molecules of NP_NTD_ (cyan and blue), 2 molecules of VP24 (orange and purple), and unassigned densities most likely corresponding to NP_CTD_ and VP35 (light gray). (**D**) Structure of recombinant EBOV NC obtained from virus-like particles composed of NP, VP35, VP24, and VP40. Top: longitudinal presentation of the helix; bottom: cross-section of the helix. The boomerang-shaped protrusions are highlighted in purple, and small protrusions are highlighted in orange. The inner layer composed of NP_NTD_ is shown in gray. Figures are modified from Wan and colleagues [15]. Figure was created with BioRender.com. Cryo-ET, cryo-electron tomography; NC, nucleocapsid.

The current structural model of the filoviral NC is based on data obtained by transmission electron microscopy (EM), cryo-electron microscopy (Cryo-EM), cryo-electron tomography (Cryo-ET), and 3D reconstruction methods, such as subtomogram averaging and mapping of available crystal structures onto obtained electron densities (Fig 2) [15].

The main determinant of the structure of filovirus NCs is the nucleoprotein NP, whose homo-oligomerization results in the formation of helical structures [6,16,17]. Transmission electron microscopy analyses of ectopically expressed NP showed that the hydrophobic N-terminal domain of NP (for EBOV amino acids 1–450 and for MARV amino acids 1–394, NP_NTD_) represents the minimal structural component responsible for NP oligomerization. NP_NTD_ oligomerization results in the formation of helices with a diameter of 28 nm corresponding to the inner ring of cross-sectioned NCs [18–21]. Cryo-ET of NP_NTD_ helices and averaging techniques revealed that the first 450 N-terminal amino acids of NP are divided into an N-terminal and a C-terminal lobe connected by a flexible hinge region (Fig 2A). A positively charged binding cleft in NP_NTD_ mediates the binding of the negatively charged RNA by electrostatic interactions [15,22–26]. Each NP monomer in the left-handed NP helix covers 6 nucleotides of the genome. The length of MARV virions is 876 nm, and that of EBOV virions is 1,028 nm; however, the sizes of their genomes are similar, namely 19,111 and 18,961 bases, respectively [25,27,28]. The different lengths of the MARV and EBOV virions are the result of the different amounts of NP molecules per rung of the NC helix leading to the differences in the diameters. The inner diameter of the MARV NC is 32 nm, which corresponds to 29.6 NP molecules. In the case of EBOV, the inner diameter of the NC helix is smaller than that of MARV, namely, 28 nm, and corresponds to an average of 24.6 NP molecules per rung. Thus, EBOV NCs have to be longer to package the whole genome. Therefore, the different diameters and corresponding lengths of the NCs determine the lengths of the viral particles [25,27,28].

The NP helix is stabilized by bound RNA as well as by interactions between the N-terminal arm-region of one NP molecule (amino acids 1–24 for EBOV and 1–19 for MARV) and the C-terminal lobe of the adjacent NP molecule within one rung of the helix (Fig 2A). Inter-rung contacts between NP molecules are formed between the C- and N-terminal regions of NP_NTD_ (Fig 2B) [15,24–26].

The hydrophilic C-terminal domain of NP (NP_CTD_) consists of an intrinsically disordered linker region and a structured tail region (NP_Ct_) that extends from the inner helix formed by NP_NTD_ [27–32]. It has been shown that NP_CTD_ disrupts the tight condensation of the NP_NTD_ helix leading to formation of loosely coiled helices [27]. Recent analysis using molecular dynamics simulation revealed different stabilities of NP-NP interactions throughout the helix, suggesting high flexibility, especially at the ends of the helix [33]. While NP_NTD_ is relatively conserved among the family *Filoviridae*, NP_CTD_ is more divergent, especially in the tail region. NP_CTD_ has been shown to mediate important interactions with other viral and cellular proteins [34–39].

**Fig 3:**
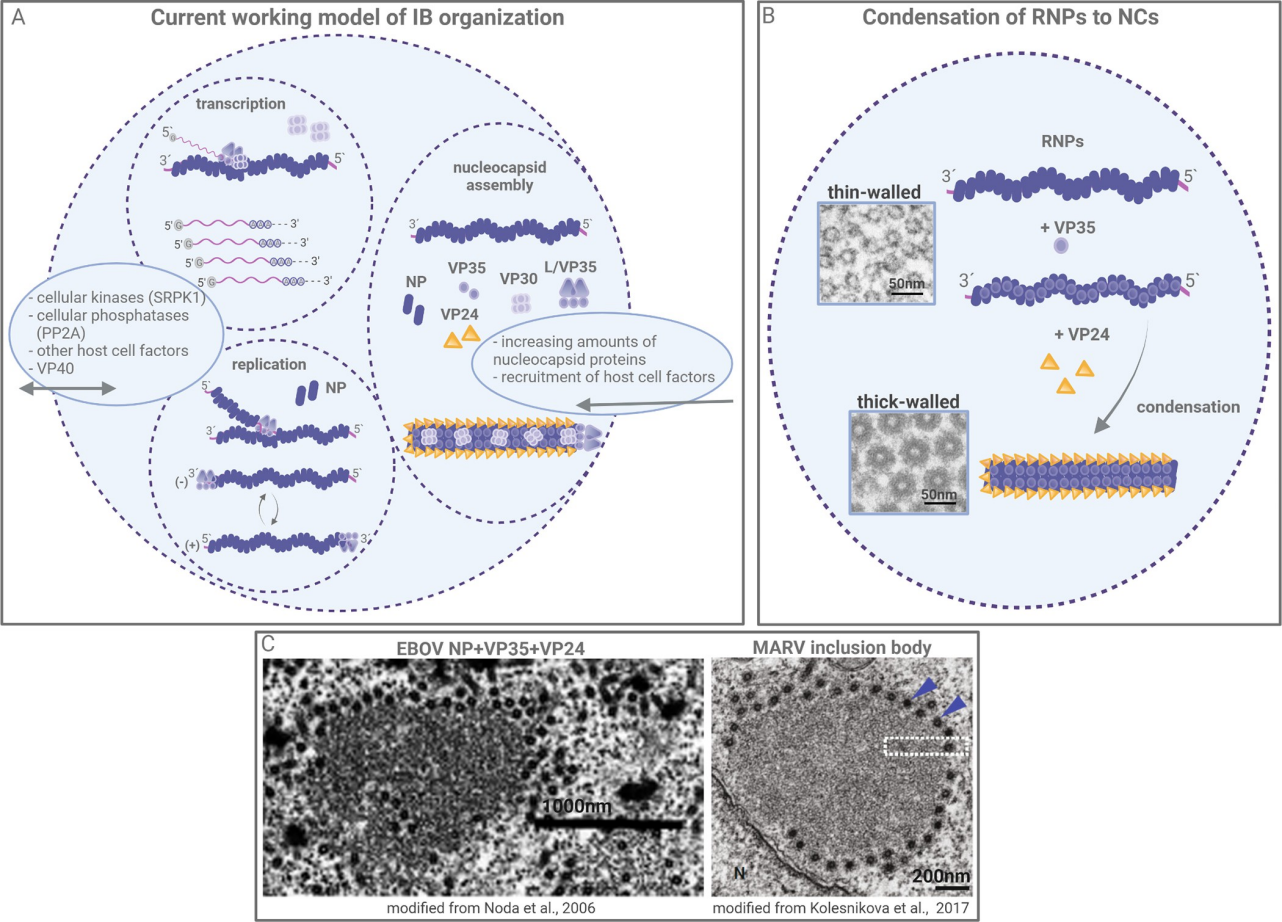
Filovirus inclusion bodies. (**A**) Current working model of IB organization. Transcription and genome replication occur inside IBs. It is possible that the 2 activities are separated in different subcompartments which, however, remains to be investigated. Possible subcompartmentalization of IBs might result from liquid–liquid phase separation based on RNA and protein concentrations. Regulation of viral transcription is partially based on the phosphorylation status of VP30, which modulates interactions with the polymerase cofactor VP35 and the polymerase L. VP30 phosphorylation is regulated by host cell kinases and phosphatases whose localization and availability might be interdependent. Increasing levels of NC proteins and possibly the recruitment of VP40 and/or host cell factors into IBs is thought to trigger the assembly of NCs. (**B**) Model of RNPs condensation to NCs in IBs. Electron micrographs from Noda and colleagues [20] show thin-walled helices upon NP expression and thick-walled helices upon coexpression of NP+VP35+VP24. The thin-walled helices detected inside IBs may represent RNPs that are active in transcription or replication or may represent intermediates of assembly. (**C**) Left picture: Electron micrograph from Noda and colleagues [20] of an IB (left) upon coexpression of EBOV NP, VP35, and VP24 showing cross-sectioned NCLS. Right picture: Electron micrograph from Kolesnikova and colleagues [14] of an IB from a MARV-infected cell with cross-sectioned NCs. Blue arrowheads indicate condensed thick-walled NCs at the periphery of the IB. Figure was created with BioRender.com. IB, inclusion body; NC, nucleocapsid; NCLS, NC-like structure; RNP, ribonucleoprotein complex.

In the presence of VP35 and VP24, the loosely coiled NP-helices change to condensed and rigid helical structures (Fig 3B) [18,20,27]. Immunocryo-EM analyses of the helical NC within filoviral particles revealed the positions of the NC-associated proteins NP, VP35, and VP24 on vertical slices of NCs, showing that in EBOV, NP forms an inner layer with a diameter of approximately 28 nm that resembles the helix formed by NP_NTD_. VP35 and VP24 were observed outside the inner NP layer [27,28,31]. Recent detailed Cryo-ET analyses combined with subtomogram averaging revealed that the overall structure of the NC inside the viral particles is a tube-like cylinder composed of aligned NP_NTDs_ with boomerang-shaped protrusions consisting of NP_CTD_, VP35, and VP24 (Fig 2C) [15,27,31]. Consistent with these observations, the ectopic coexpression of NP, VP35, and VP24 resulted in the formation of NC-like structures (NCLS) with the same symmetry and structure as the NCs in virions but with more variable lengths (Fig 2D) [15,27,31]. The localization of the functional polymerase complex consisting of VP35 and L and that of the transcription factor VP30 in the viral NC are thus far unresolved, possibly because these proteins are scattered over the NC. Interestingly, Cryo-ET revealed that the NCs possess a pointed and a barbed end, which complemented the observation that NC budding is always directional with the pointed end in front [27,28,40].

## 3. Inclusion bodies as de novo nucleocapsid assembly sites

### 3.1. IBs formation

A hallmark of filovirus-infected cells is the formation of IBs, which, in EM analyses, appear as the accumulation of thin-walled, apparently flexible helices and more rigid thick-walled, electron-dense helices embedded in a matrix of viral and cellular proteins (Fig 3C) [6,7,20,41,42]. For many years, whether IBs performed a function in the viral replication cycle or whether IBs represented dead-end accumulations of viral material remained under debate. Investigations in the last decade, however, revealed that viral IBs represent membrane-less virus-induced compartments with high structural and functional dynamics that are similar to cellular membrane-less compartments called liquid organelles [43–45]. Although not systematically proven, it is very likely that filovirus IBs also represent liquid or liquid-like organelles formed by liquid–liquid phase separation, similar to IBs of other mononegaviruses as reviewed in [46]. For example, disassembly and reassembly of IBs in EBOV-infected cells during cell division is a typical feature of liquid organelles and has been shown in live-cell imaging experiments [11,46]. Further live-cell imaging experiments revealed the shape of IBs to be highly dynamic and their size is also determined by fusion and fission events [9,10]. It was previously shown that transient expression of filovirus NP on its own could induce the formation of IBs [6,47,48]. Truncation of NP at the N-terminus or the C-terminus resulted in inhibition of IBs formation [35,36]. Interestingly, coexpression of VP35 with NP constructs that were unable to form IBs (NP_ΔCt_) can rescue IBs formation, suggesting that the interaction of NP and VP35 can control the early steps of this process [35]. Both NP and VP35 share characteristic properties of proteins that form liquid organelles, such as intrinsically disordered domains, multivalency, phosphorylation, and RNA-binding ability [43]. Liquid–liquid phase separation might be necessary to create a protected environment inside the infected cell to enable replication and transcription as well as NC assembly [49].

### 3.2. NC-assembly steps in the IBs

Currently, the precise order of the processes leading to the formation of NCs inside of IBs is still unknown. Similarly, while transcription and replication of filovirus RNA take place inside IBs, it is not clear how these 2 processes are separated in time and space from NC assembly (Fig 3A) [11,12]. Based on the available data, we suggest the following model of RNP formation and NC assembly (Fig 3A and 3B).

It has been shown that an N-terminal peptide of VP35 functions as a chaperone that maintains NP in a monomeric state before the oligomerization of NP and encapsidation of the viral RNA occurs concomitantly to replication [50–52]. The interaction of the 2 proteins is complex and involves several interaction domains on NP and VP35 [13,42,47,50,51,53,54]. Although it is currently unknown when during the viral RNA synthesis VP35 is recruited to the NP-RNA complex, a stoichiometric ratio of the 2 proteins has been shown to be crucial for the formation of thin-walled helices that are detected in the filoviral IBs [20,42]. Electron microscopy so far was not able to distinguish thin-walled helices composed of NP-VP35-RNA or NP-RNA. It is also not known which of the 2 complexes serves as template for RNA synthesis by the viral polymerase (Fig 3B and 3C). Similar to other mononegaviruses, the functional polymerase composed of VP35 and L is recruited to IBs via the interaction between NP and VP35 to enable RNA synthesis [8,11,12,53,55–57]. The positions of the polymerase complex inside NCs in not known so far. It has been shown that VP30 is recruited into IBs by direct interactions with NP; however, its position in the NC is also unresolved [50,58–61].

### 3.3. Separation of transcription and replication in IBs

While replication of filoviral genomes requires the presence of NP, VP35, and L, transcription is dependent on the transcriptional initiation factor VP30 [62]. The transcriptional support activity of VP30 is switched off upon phosphorylation. Thus, phosphorylation of VP30 regulates the balance between transcription and replication [63–65]. Phosphorylation of VP30 also regulates its interaction with NP and is mediated by the cellular kinase SRPK1, and dephosphorylation is catalyzed by PP2A. Both the phosphorylation and dephosphorylation of VP30 occur in IBs, demonstrating the importance of host cell factors for the regulation of viral mRNA transcription and replication in IBs [39,59,65]. Additionally, it was shown that the interaction of VP30 with the polymerase cofactor VP35 modulates transcription and that VP30 can interact directly with L [48,64]. Although it is not known exactly how the switch from transcription to replication is mediated, the current data suggest that posttranslational protein modifications, such as phosphorylation, influence protein–protein, and/or protein–RNA interactions and thus regulate this process (Fig 3A) [63,64]. Increasing levels of NP and VP35 and possibly increasing levels of phosphorylated VP30 in IBs may also activate replication of the viral genome. Further in vitro transcription/replication studies are needed to answer these remaining questions.

It is currently unknown how the transcription and replication of viral RNA are temporally and spatially organized inside filoviral IBs. An interesting observation made by Nelson and colleagues in EBOV-infected cells already indicated potential IBs subcompartments when distinct granules were detected within IBs containing EBOV NP mRNA together with eIF3 and other stress granule-associated (SG) proteins [66]. Sequestration of SG-associated proteins into IBs has been suggested to interfere with the activation of the innate immune system [67–69]. Interestingly, for respiratory syncytial virus (RSV), transient formation of granules inside IBs was observed that contained viral mRNA and the viral transcription anti-terminator protein M2-1 but not the other viral proteins involved in RNA synthesis, the nucleoprotein N, phosphoprotein P or L [70]. It remains to be investigated if filovirus IBs possess an internal organization that leads to transient sequestration of viral mRNA as well. Likewise, the details of spatiotemporal organization of filovirus transcription and replication and NC maturation has yet to be resolved (Fig 3A).

### 3.4. RNP condensation to NCs

The maturation of thin-walled helices (RNPs) into thick-walled helices (corresponding to NCs) can only be observed in the presence of VP35 and VP24, and thick-walled helices are detected mainly in the periphery of IBs (Fig 3B and 3C) [16,18–20,36]. High levels of VP24 inhibit transcription and replication, suggesting that increasing levels of VP24 in IBs block viral RNA synthesis in favor of NC maturation [71–76]. VP24 is recruited into IBs by interaction with NP [16,19,20,71,72,74,75]. Recently, it was demonstrated that EBOV VP24 can be SUMOylated and ubiquitinated and undergoes nucleocytoplasmic shuttling [77,78]. Whether posttranslational modification regulates the recruitment of VP24 into IBs and/or its functions in NC condensation and transport remains to be determined.

## 4. Transport of nucleocapsids from inclusion bodies to budding sites

The ectopic expression of fluorescently labeled VP30 (VP30-GFP) in EBOV- or MARV-infected cells results in fluorescently labeled NCs, whose movement can be monitored by live-cell imaging. Using this approach, the ejection of single NCs from IBs could be observed, and their intracellular transport to the plasma membrane could be tracked and characterized in living cells [9,10].

**Fig 4:**
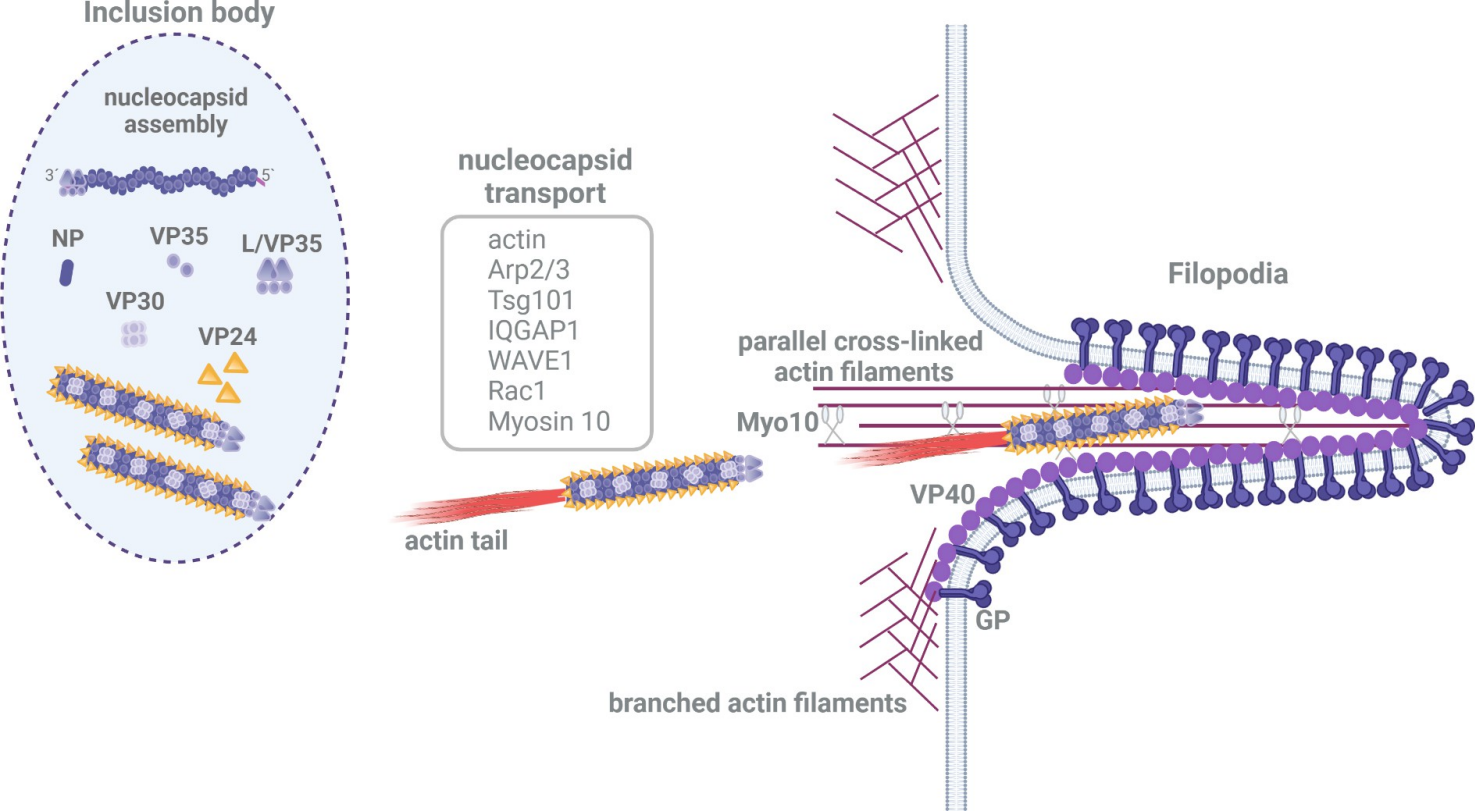
Transport of nucleocapsids from inclusion bodies to budding sites. Transport-competent NCs composed of all the NC proteins are formed inside IBs. The involvement of different host cell factors is required for the actin-dependent transport of NCs (gray box). Outside the IBs, actin tails are formed at one end of the NC in the cytosol, which drive their transport. After reaching the plasma membrane, budding of filoviruses occurs mainly at filopodia, in which myosin 10 may support the transport of NCs along parallel actin filaments. Figure was created with BioRender.com. IB, inclusion body; NC, nucleocapsid.

Since the long filovirus NC cannot reach the budding site by diffusion alone, interactions with host cell factors are required to mediate its transport. The application of cytoskeleton-depolymerizing drugs revealed that the transport of filovirus NCs is driven by an actin-dependent mechanism [9,10,79,80]. Other intracellular pathogens, such as *Listeria monocytogenes* or baculovirus, express proteins that directly interact with actin and the actin polymerization complex Arp2/3 to promote the polymerization of branched actin filaments [81]. Such nucleation-promoting factors are required for the activation of the Arp2/3 complex to mediate actin polymerization [81,82]. Direct interactions between filovirus NC proteins and actin or the Arp2/3 complex have not yet been described. To promote actin polymerization for their transport, filovirus NCs may therefore recruit cellular nucleation-promoting factors, which provide a link to actin and the Arp2/3 complex (Fig 4).

Using specific inhibitors and silencing host cell factor expression by siRNA, it was demonstrated that the Arp2/3 complex, WAVE1 and Rac1 play important roles in the actin-dependent transport of NCLS and authentic viral NCs [9,10,83]. Moving filoviral NCLS and NCs display actin comet tails at one end, suggesting a transport mechanism previously described for the intracellular transport of vaccinia virus and baculovirus capsids that is based on the polymerization of branched actin filaments [81,84]. The NP of MARV was shown to recruit cellular factors into the IBs, such as Tsg101 and IQGAP1, a regulator of actin dynamics and filopodia formation, which is also involved in regulating the transport of NCs. Down-regulation of Tsg101 or IQGAP1 expression affected the transport of NCs, resulting in their accumulation close to the plasma membrane and reduced budding at filopodia suggesting different transport steps of NCs from the IBs at first to the planar plasma membrane and then further into filopodia [85]. Filopodia that are enriched with VP40 are the preferred budding sites of filoviruses [9,10,85,86]. This is consistent with data showing that EBOV VP40 strongly enhances the recruitment of NCLS into filopodia [79]. Filopodia are long, thin cellular protrusions containing characteristic parallel actin filaments that are cross-linked by fascin [87]. Unconventional myosin 10 mediates the transport of cargo along actin filaments within filopodia. Myosin 10 was shown to transport VP40, and it is hypothesized that NCs make use of this mechanism for transport inside filopodia (Fig 4) [9,10,86,88]. The exact molecular interactions between actin polymerization complexes that drive the actin-dependent transport of the NC and the viral NC proteins remain to be determined. Using a minimalistic live-cell imaging system based on fluorophore-tagged NC proteins, it was shown that NP, VP35, and VP24 are essential and sufficient for forming electron-dense and transport-competent helical NCLS [16,18–20,36,71,79,80]. Mutation of the YxxL motif of VP24 in EBOV resulted in NCLS that exhibited significantly impaired transport by an unknown mechanism [89]. Taken together, the current literature demonstrates that both NP and VP24 seem to regulate the actin-dependent transport of NCLS via different mechanisms. Further experiments with the minimalistic live-cell imaging system and application of advanced NC-tracking software are needed to define the molecular mechanism underlying the formation of the actin tail at one end of NCLS that drives their transport.

Different transport kinetics were described for filovirus NCs depending on their intracellular location. The velocities range from 100 nm/s in the filopodia to 400 nm/s in the cytosol, and these differences suggest the involvement of different actin-based transport machineries during transport from IBs to budding sites [9,10,79,80]. The molecular details of the observed differences in the transport velocities of NCs are still unclear. In addition to actin tail-driven transport, NCs were observed to move along actin filaments, suggesting motor protein-dependent and motor protein-independent transport mechanisms [10]. For measles virus, the transport of RNPs from IBs to the plasma membrane is dependent on actin filaments, whereas their incorporation into new virions requires actin dynamics at the cell periphery [90]. Transport kinetics and cytoskeleton inhibitor studies of NCs of other mononegaviruses, such as vesicular stomatitis virus, revealed the involvement of microtubule- and actin-dependent transport mechanisms [91]. Most likely, microtubule-dependent transport does not play a major role in the transport of filovirus NC [9,10,79,80].

The formation of an actin tail at one end of the filovirus NC resulted in directional transport and budding of the particles with the pointed end of the NC in front. It is unknown how the binding of the actin polymerization machinery to one end of the NC is regulated. For example, it was shown that positioning the baculoviral proteins p78/83 or VP80 at one end of the NC influenced the directionality of transport [92–94]. Thus, the structural polarity of the filovirus NC may be the result of the recruitment of the cellular actin polymerization machinery to the barbed end, since budding was shown to occur with the pointed end first (Fig 4) [28,40]. It is tempting to hypothesize that the position of the viral polymerase complex in the NC at the 3′ end of the genome, where it can initiate primary transcription as shown for other mononegaviruses, may influence the recruitment of transport machinery [95,96]. For vesicular stomatitis virus, however, super-resolution imaging revealed that the viral polymerase is located at the blunt end of the bullet-shaped particles where the 5′ end of the genome is located [97]. The viral determinants responsible for recruitment of the actin polymerization machinery at one end of the NC remain to be identified using super-resolution techniques and live-cell imaging.

Interactions of the matrix protein VP40 with NP in the IBs inhibit viral transcription and replication possibly by partial NC condensation and on the other hand enable NC envelopment at the plasma membrane and budding [27,38,73,98,99]. Live-cell imaging studies of MARV-infected cells revealed that outside of IBs association of VP40 with NCs was detected only at the plasma membrane, arguing against a contribution of VP40 to the transport of NCs from IBs to the plasma membrane [10]. The incorporation of NCLSs into infectious virus-like particles (also referred to as transcription and replication competent virus-like particles, trVLPs) at the plasma membrane was dependent on tyrosine phosphorylation of VP40, which occurs at cellular membranes, indicating that docking of NCLS to the plasma membrane and envelopment are regulated by the posttranslational modification of VP40 [98].

## 5. Conclusions and perspectives

Current data suggest that NP is a highly dynamic protein that organizes protein–protein and protein–RNA interactions to promote RNP and NC formation. The resulting helical structure with boomerang-shaped protrusions has a pointed and a barbed end, generating structural asymmetry. The complete 3D structure showing the location of all NC proteins remains to be solved, which will require high-resolution Cryo-EM and Cryo-ET studies and structure reconstruction methods based on authentic viruses and trVLPs.

The assembly of NCs occurs in NP-induced IBs, which are likely liquid-like organelles. Posttranslational modifications of viral NC proteins, such as phosphorylation and interactions with host cell factors, seem to regulate their formation. Techniques used in liquid organelle biology have to be applied to investigate the liquid–liquid and liquid–solid phase transitions in IBs formation and to further dissect the role of the different viral and cellular factors involved.

NP, VP35, and VP24 are sufficient and essential for forming mature and transport-competent NCs, which require the recruitment of actin polymerization factors for transport from the IBs to budding sites. RNA labeling techniques, multicolor live-cell imaging, and super-resolution imaging and techniques such as correlative light electron microscopy can help to identify the molecular determinants of the viral and host factors that are involved in the formation and transport of NCs.
